# Hyperloop-like diffusion of long-chain molecules under confinement

**DOI:** 10.1038/s41467-023-37455-3

**Published:** 2023-03-28

**Authors:** Jiamin Yuan, Mingbin Gao, Zhiqiang Liu, Xiaomin Tang, Yu Tian, Gang Ma, Mao Ye, Anmin Zheng

**Affiliations:** 1 State Key Laboratory of Magnetic Resonance and Atomic and Molecular Physics, National Center for Magnetic Resonance in Wuhan, Wuhan Institute of Physics and Mathematics, Innovation Academy for Precision Measurement Science and Technology, Chinese Academy of Sciences, Wuhan, 430071 People’s Republic of China; 2 University of Chinese Academy of Sciences, Beijing, 100049 People’s Republic of China; 3 National Engineering Laboratory for Methanol to Olefins, Dalian National Laboratory for Clean Energy, iChEM (Collaborative Innovation Center of Chemistry for Energy Materials), Dalian Institute of Chemical Physics, Chinese Academy of Sciences, Dalian, 116023 People’s Republic of China; 4 College of Chemistry and Materials Science, Hebei University, Baoding, 071002 People’s Republic of China

**Keywords:** Heterogeneous catalysis, Chemical physics, Chemical physics

## Abstract

The ultrafast transport of adsorbates in confined spaces is a goal pursued by scientists. However, diffusion will be generally slower in nano-channels, as confined spaces inhibit motion. Here we show that the movement of long-chain molecules increase with a decrease in pore size, indicating that confined spaces promote transport. Inspired by a hyperloop running on a railway, we established a superfast pathway for molecules in zeolites with nano-channels. Rapid diffusion is achieved when the long-chain molecules keep moving linearly, as well as when they run along the center of the channel, while this phenomenon do not exist for short-chain molecules. This hyperloop-like diffusion is unique for long-chain molecules in a confined space and is further verified by diffusion experiments. These results offer special insights into molecule diffusion under confinement, providing a reference for the selection of efficient catalysts with rapid transport in the industrial field.

## Abstract (web summary)

Normally, the diffusion of the object is suppressed in the confined space. Here, by regulating the degrees of freedom of the molecule, authors demonstrate ultrafine diffusion in the sub-nano space.

## Introduction

Rapid transport in sub-nanometer channels has been a major challenge in the industry for many years^1–5^, as confined spaces will inhibit diffusion under normal circumstances^3,6,7^. Studies have shown that confined channels at the sub-nanometer level were important vessels for catalysis^8–11^. Adsorbates under confined conditions were shown to exhibit different properties compared to their corresponding bulk phases, such as miscibility, phase transitions, and diffusion^12^. Many studies have revealed that diffusion under confinement will be more than an order of magnitude slower than in the bulk phase^13–15^, demonstrating that strong confinement will always be accompanied by slow diffusion. Although the diffusion and mass transfer performance of porous materials will significantly affect their catalytic efficiency and selectivity^8^, no efficient solutions are currently available to obtain rapid diffusion in strongly confined spaces (especially for bulky or long-chain molecules). Therefore, ultrafast diffusion in confined spaces is an urgent problem, as well as a popular and challenging research topic in catalysis, separation, and other fields^16–19^.

Long-chain alkanes are important components of diesel (C10–C20) and lubricants (C20–C40), and are obtained from naphtha catalytic cracking by the zeolite catalysis^20^. In this process, the catalytic performance is strongly dependent on alkane diffusion property inside microporous zeolites^21^. Therefore, the study of the diffusion behavior of these alkane molecules in zeolite channels is of great value for basic research and industrial applications. The pioneering work by Derouane and Yashonath demonstrated fast diffusion by matching the channel size and dimension of monoatomic or small molecules with less freedom (in this case, the dimension is almost fixed)^20–24^. It is noteworthy that due to the large freedom of long-chain alkanes (i.e., C12 in our work), molecular size is always varied in large ranges (i.e., larger than 6 Å) due to their great flexibility coupled with their rotational dynamics and unpredictable shape. Therefore, it is a challenge to realize the matching conditions of levitation diffusion between channel size and molecular size proposed by ref. ^22^ and refs. ^23–26^.

**Fig. 1:**
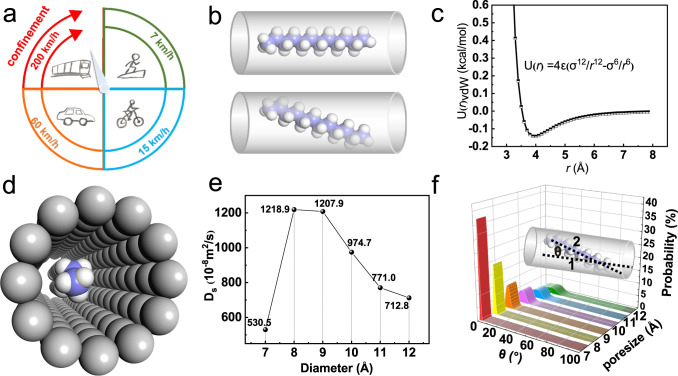
Hyperloop-like diffusion under confinement. **a** Examples of movement in daily life. **b** Schematic showing n-dodecane (C12) molecule transport in zeolite channels on the right track (top) and deviating from the track (bottom)**. c** Van der Waals interactions between C12 and zeolite. **d** Stereogram model of the C12 molecule in the nanometer channel. **e** The diffusion coefficients of C12 in the confined channel with various diameters. **f** Deviation angle between the axial direction of the channel (inset line 1) and the end-to-end line of the C12 molecule (inset line 2). (Purple and white spheres represent the carbon and hydrogen atoms of alkane, and gray balls in Fig. 1d represent the framework of sub-nano channel). Source data of Fig. 1f is provided as a Source Data file.

As art comes from life (Fig. 1a), macroscopic motion and confinement have always existed. For example, humans can walk on rugged mountain roads at a very low speed, while bicycles drive faster on curved roads. Although cars offer fast transport, they can only be driven on wide roads. For superfast high-speed trains, their requirements are even higher, as they can only run on railroad tracks. Thus, it appears that the faster the speed, the higher the track requirements (confinement). Subsequently, we can turn our attention to the hyperloop, a type of transportation with vacuum steel tube transportation (confined space) as its core theory, as well as with the characteristics of ultra-high speed, high safety, and low energy consumption. Inspired by the working principle of the hyperloop, long-chain alkane transport inside zeolite channels can be analogous to a high-speed train running on the track. In this case, the effective manipulation of long-chain molecules suspended in the centers of zeolite channels and the maintenance of parallel motion to the channel were very important for achieving ultrafast diffusion in confined channels (Fig. 1b top), while the off-track motion would significantly slow down the movement (Fig. 1b bottom). In this work, an idealized model for long-chain alkane ultrafast diffusion was estimated; the most suitable zeolite channel for n-dodecane (C12) transportation was screened out. Finally, ultrafast diffusion performance was confirmed by additional diffusion experiments.

## Results

The diffusion behavior of molecules inside confined spaces was strongly affected by channel^4,9,27,28^. Based on the van der Waals analysis (Fig. 1c and Supplementary Fig. 1: see Supplementary Note 1 for details), we built a series of sub-nanometer channels with various interior diameters (from 7 to 12 Å as the superfast track) for the n-dodecane (C12) molecule (regarded as the train) diffusion (Fig. 1d). The details of model construction are described in the Supplementary Note 1. Subsequently, molecular dynamics (MD) simulations were carried out to study the diffusion performance. To quantitatively analyze the movement of guest molecules inside the confined channels, a self-diffusion coefficient (D_s_) was calculated^3,28^. As shown in Fig. 1e, the D_s_ in these models showed superfast values (the D_s_ values of C12 were 530.4, 1218.9, 1207.9, 974.7, 771.0, and 712.8 × 10^−8^ m^2^/s in channels with a pore size of 7 to 12 Å), which was even larger than the C12 molecules in the liquid phase (0.13 × 10^−8^ m^2^/s). Indeed, a diffusion maximum occurred at the 8 Å channel, and among all of these models, both the smaller (e.g., ≤7 Å) and larger (e.g., ≥9 Å) channels would slow down diffusion due to repulsion or attraction interactions. In the channels with small pore size, strong repulsion to the adsorbate would decrease the diffusion coefficient, while in a larger space, the attraction of the channel wall would cause the molecules to deflect (like a derailed train) and decelerate the movement. Based on the quantitative analysis of the deviation angle (Fig. 1f) with different peak values (e.g. 2.0, 3.0, 4.5, 7.5, 12.0, and 17.0 degrees when the pore size ranged from 7 to 12 Å, respectively) and location (Supplementary Fig. 4), we found that the larger the pore size, the easier it was for long-chain molecules to deviate from the original direction of travel. Overall, ultrafast diffusion could be achieved by regulating the pore size of the confinement channel.

**Fig. 2:**
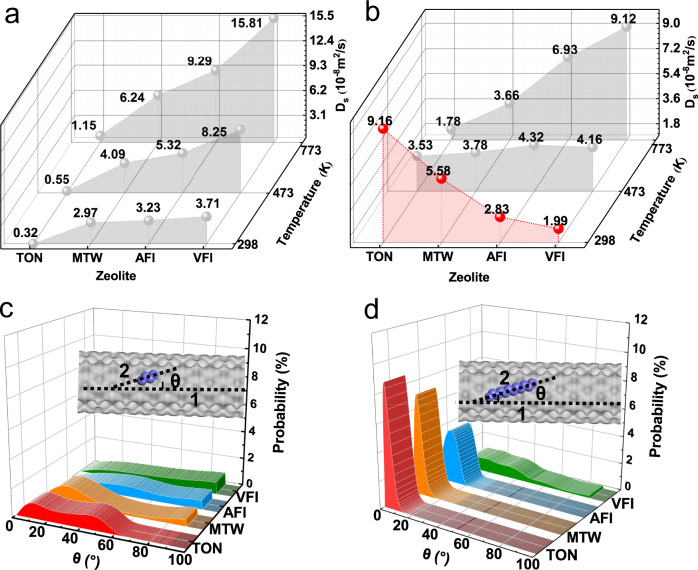
Self-diffusion coefficients and the deviation angles for the alkane molecules in the zeolites. The self-diffusion coefficients of **a** n-butane (C4) and **b** n-dodecane (C12) in the 1D channel zeolite at various temperatures (The gray balls represent the data points). The deviation angle between the axial direction of the zeolite channel (inset line 1) and the end-to-end line of the **c** C4 molecule (inset line 2) and **d** C12 molecule at 298 K (purple spheres represent the carbon atoms). Source data of Fig. 2a–d are provided as a Source Data file.

Zeolites, widely used as catalysts in the industry^6,29,30^, and with various pore sizes in the channel from a sub-nanometer to the nanometer scale, were a possible suitable candidate for superfast transport. Therefore, methane, n-butane, n-octane, and n-dodecane molecules (the main components in natural gas, gasoline, and diesel^31,32^) were chosen as the main adsorbates. In addition, four types of one-dimensional zeolites with TON (7.3 × 8.4 Å^2^), MTW (8.3 × 8.7 Å^2^), AFI (10 × 10 Å^2^), and VFI (15.4 × 15.4 Å^2^) topologies were selected as the main research objects (Supplementary Fig. 2). As shown in Fig. 2a, b, the D_s_ of the alkanes in the zeolites were calculated and were in the range of 10^−9^ to 10^−8^ m^2^/s. For example, the D_s_ of n-butane (C4) in the TON zeolite was 0.32 × 10^−8^ m^2^/s at 298 K, which was in accordance with Schuring’s work^33^ (0.22 × 10^−8^ m^2^/s at 333 K). The D_s_ of methane (C1) in AFI (2.65 × 10^−8^ m^2^/s) was similar to that reported by ref. ^34^ (about 2.70 × 10^−8^ m^2^/s). Consistent with the results calculated by ref. ^34^ (2.8 × 10^−8^ m^2^/s), the calculated D_s_ of methane in MTW was 2.32 × 10^−8^ m^2^/s at 298 K in this work (Supplementary Fig. 5). Figure 2a shows that the D_s_ of C4 showed a gradual increase with an increasing pore size of the zeolite channel. The D_s_ values were 0.32, 2.97, 3.23, and 3.71 × 10^−8^ m^2^/s for the TON, MTW, AFI, and VFI zeolites at 298 K, respectively. Of note, in contrast to C4, counterintuitive behavior was observed with a steep drop in the D_s_ of long-chain alkane C12, along with increasing pore size (Fig. 2b) at 298 K. For example, the D_s_ values of C12 in TON, MTW, AFI, and VFI were 9.16, 5.58, 2.83, 1.60 × 10^−8^ m^2^/s respectively. In addition, we also considered other molecules, such as n-cetane, which presented the hyperloop-like diffusion under confinement (9.34, 6.80, 2.62, and 1.76 × 10^−8^ m^2^/s in TON, MTW, AFI, and VFI, respectively) at 298 K. While the anomalous phenomenon mentioned above was not observed at higher temperatures and will be discussed in later sections. In summary, the movement of long-chain molecules increased with decreasing pore size, indicating that the confined space promoted transport.

To understand the two above-mentioned phenomena with completely opposite trends of diffusion with pore size, the confinement effect of zeolite channels on the alkane molecules had to be analyzed^8,35^. The reduced density gradient (RDG)^36^ (Supplementary Fig. 6) shows that there was a greater confinement effect in the zeolite with small pore size. Based on the RDG, it appeared that long-chain molecules preferred to undergo almost linear diffusion along the center of the TON zeolite, as slight deviation caused strong repulsion. When the pore size increased (i.e., in MTW, AFI, and VFI), it was harder to maintain transport in the center with the existence of confinement^37^, which could apparently enhance the possibility of collisions between the molecules and zeolite channels. Thus, it could dramatically slow down the diffusion. We subsequently presented a quantitative analysis of the relative position between the molecule and the zeolites. As shown in Fig. 2d and Supplementary Fig. 7, the deviation angle between the axis of the channel and the C12 molecule (Supplementary Fig. 3) was proportional to the pore size. For example, the peak values of this angle were located at 3.5, 4.5, 10.0, and 19.5 degrees in TON, MTW, AFI, and VFI, respectively. Specifically, we observed that the angle distribution in VFI was more extensive (even up to 90 degrees), which meant that the molecules could move from the axis of the channel to the radial section, increasing the frequency of collisions between the molecules and channels, resulting in slower diffusion (Supplementary Movie 2). Dramatic differences in diffusion-pore size dependence between the short-chain (e.g., n-butane) and long-chain (e.g., n-dodecane) molecules at 298 K are illustrated in Fig. 2c, d. Similar to C12 in the large zeolite, C4 in the small pore size zeolite was relatively free to rotate, and was attracted to the folds on the surfaces of the channels (Supplementary Fig. 8 and Supplementary movie 3). Thus, the deviation angle of the C4 molecules in TON and MTW showed a wide distribution (Fig. 2c). Furthermore, the free energy analysis further verified that the C4 molecule preferred to locate in the curved position, while the C12 molecule preferred to appear in the center of the channel (Supplementary Fig. 8 and Supplementary Movie 1). Thus, regardless of the pore size of the channel, short-chain molecules would depart from the orbit, leading to more collisions between the molecules and zeolites. Thus, diffusion of C4 in the small pores was slower than in large pores (Supplementary Movies 3, 4).

**Fig. 3:**
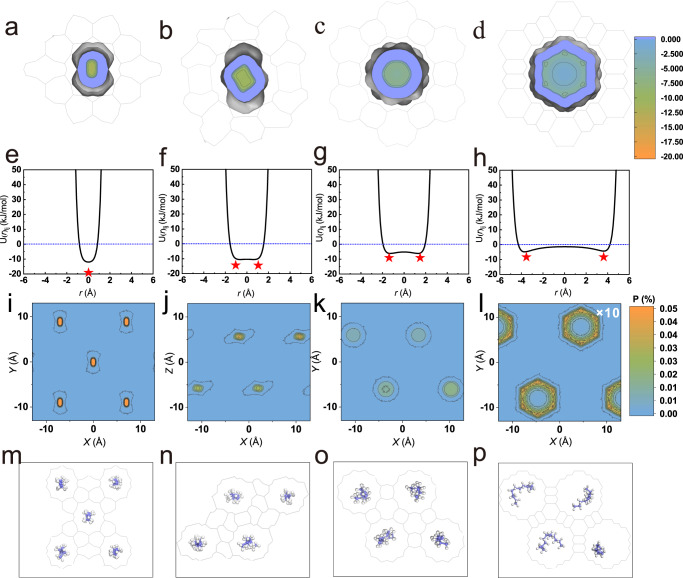
Van der Waals interactions and configuration distribution of n-dodecane (C12) in the zeolites. **a**–**d** Plane and **e**–**h** linear graph of van der Waals interaction energies between zeolite and C12 (red star indicates the minimum point, gray lines represent the framework of zeolite). **i**–**l** The probability of n-dodecane (C12) molecule distribution in the radial plane of the zeolite channel. **m**–**p** Corresponding structure diagram of a random frame of n-dodecane diffusion in zeolite, with TON, MTW, AFI, and VFI zeolite from left to right (purple and white spheres represent the carbon and hydrogen atoms).

To further determine the effect of molecule confinement by different channels, the van der Waals interactions (negative and positive values represented attraction and repulsion, respectively) between the zeolites and C12 molecules were calculated. As shown in Fig. 3a–h, the most significant differences for these zeolites were the number of adsorption sites. For example, there was only one maximum adsorption position located in the center of the small channel (Fig. 3a, e), which indicated the two strongest adsorption sites located near the channel wall in the zeolite with a large pore size (Fig. 3b–d, f–h). This meant that C12 in the small pore size channels were more likely to be adsorbed right in the center of the channel, while those in the large pore size appeared near the channel wall. The position distributions of C12 (all carbon atoms) in the radial plane of the zeolite channel in the MD simulations were statistically analyzed (Fig.3i–l and Supplementary Fig. 9), and we found that the distribution of C12 in the TON zeolite (Fig. 3i) with smaller pore size was much denser than in the zeolites with a larger pore size (Fig. 3j–l). In addition, the molecules almost did not appear in the center of the channel, but were distributed near the channel wall in VFI according to the skin effect (Fig. 3l), which was in accordance with the van der Waals analysis (Supplementary Fig. 1) of the ideal model^38^. This was further confirmed by ab initio MD simulations (Supplementary Fig. 10). A random frame of the structure during the diffusion process was extracted and is shown in Fig. 3m–p, and these results were in accordance with the trend illustrated in Fig. 3i–l, where the smaller the pore size, the more uniform the molecular distribution in the center of the channel. In addition to the position shift of the molecules in the zeolite, we also observed tiny molecular deformations with a small pore size (Fig. 3m). With an increase in pore size, the molecular deformation increased and the bending amplitude became larger (Fig. 3n–p), which increased the diffusion resistance in the zeolites with a larger pore size. Therefore, the larger position shift and deformation in the channel with a larger pore size would slow down the diffusion of the C12 molecules under confinement.

**Fig. 4:**
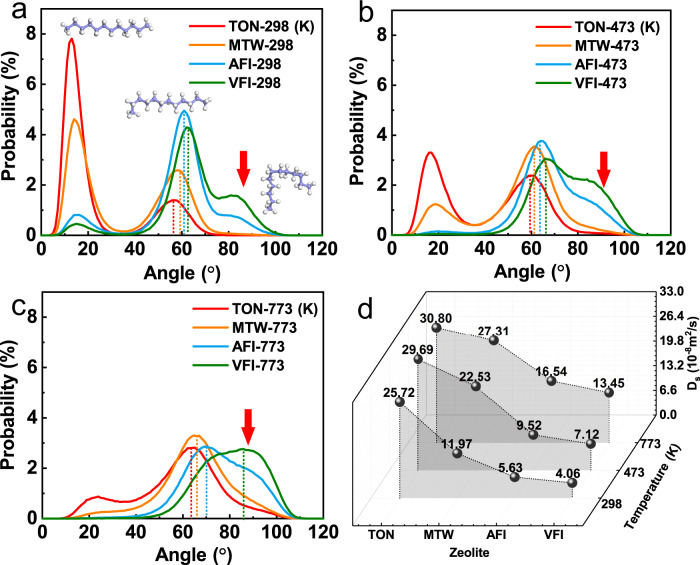
The deformation angle and diffusion coefficients in the zeolites. The deformation angle of C12 in the 1D channel of the zeolite at **a** 298 K, **b** 473 K, and **c** 773 K. **d** The diffusion coefficients of the rigid-C12 (linear chain) in the 1D channel of the zeolite. Source data of Fig. 4d is provided as a Source Data file.

Quantitative comparison of molecular deformation in the different channels further illustrated the superfast diffusion. As shown in Fig. 4a, compared to TON and MTW, the deformation curves of C12 in AFI and VFI showed an obvious peak around 85 degrees. As the temperature increased, the position of this peak shifted to a higher degree (90 degrees at 473 K, 95 degrees at 773 K), and the peaks of TON and MTW gradually emerged (Fig. 4a–c). The large bending angle of C12 destroyed the linearity of the molecule and suffered greater steric hindrance (the shape changes of C12 within the various zeolites are shown in Supplementary Fig. 11) in the zeolite with a small pore size, while it presented small steric hindrance for the deformed molecules diffusing in the zeolites with a large pore size. For verification, the C12 molecule was set as rigid (a straight linear molecule, as shown in Fig. 1b, top), and we recalculated its diffusion coefficients. The D_s_ values at different temperatures are shown in Fig. 4d, which clearly indicates that diffusion in the zeolite with a small pore size occurred faster than with a large pore size, regardless of the temperature (298, 473, and 773 K), as long as the molecule maintained linearity. Because the molecules were free to move fast along the center of the channel of the small channel (just like a train running on a rail), they slowed down and easily deviated from the center trajectory in the zeolite with a large space. Furthermore, we found that the center of the channel possessed the lowest energy barrier (Supplementary Fig. 12), demonstrating the advantage of diffusion in the center of the channel. In addition, we found that straight molecules spread out faster than curved ones (Figs. 4d, 2b). Overall, the molecules located in the center of the channel, as well as those maintaining linearity, promoted diffusion under confinement.

**Fig. 5:**
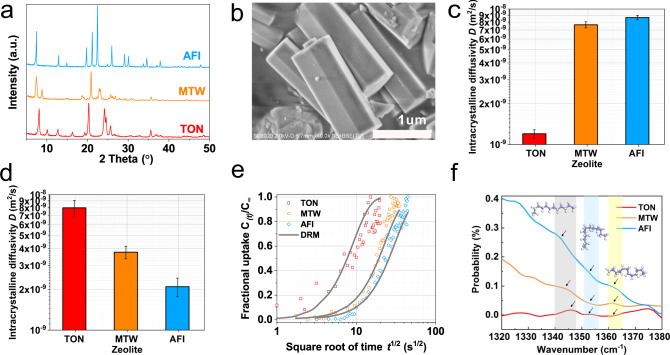
Experimental results for C4 and C12 diffusion in the zeolites. **a** The XRD patterns of the TON, MTW, and AFI zeolites. **b** SEM pictures of the AFI zeolite, **c** The uptake curve and intracrystalline diffusivity of **d** C4 and **e** C12 (error bar represents the experimental fitting error). **f** Infrared spectroscopy of the n-dodecane molecules in the different zeolites at 298 K (The gray, blue, and yellow areas indicate the peaks corresponding to the configurations in the image, and the arrows help guide the view).

Diffusion experiments were conducted to further verify ultrafast diffusion. The uptake rate measurements of C12 in the TON, MTW, and AFI zeolites at room temperature (298 K) were carried out by an in situ infrared microscope^39,40^. The powder X-ray diffraction (pXRD) patterns and scanning electron microscope (SEM) images of the zeolite samples are shown in Fig. 5a–b and Supplementary Fig. 13, respectively (see Supplementary Table 1 for more crystal parameters). The intracrystalline diffusivity D (Fig. 5c, d) was calculated by decoupling the uptake curve^41^ (Fig. 5e), which was obtained by integral IRM mode (wavenumbers in the range of 1350–1620 cm^−1^, Supplementary Fig. 14). The diffusion trend of the crystal obtained by this method was in good agreement with the theoretical calculations, indicating that the larger the pore size, the slower the diffusion of C12. In addition, diffuse reflectance infrared Fourier transform (DRIFT) spectroscopy^42^ of the n-dodecane molecules in the different zeolites also demonstrated that more molecular deformation occurred inside the larger pore-sized zeolite (Fig. 5f). For instance, the majority of molecules in TON had a wavenumber near 1341 cm^–1^, which represented end-gauche^42^, while AFI had a higher peak at 1364 cm^−1^ (kink deformation). Furthermore, the diffusion coefficients of C4 (Fig. 5c) were in excellent agreement with the D_s_ calculated by the MD simulations above. This further confirmed that the hyperloop-like diffusion only exists for long-chain molecules under confinement.

## Discussion

Fast diffusion in a confined environment is a hot topic that has been extensively investigated in previous studies^23,26,43^. Both the floating molecule^22^ proposed by Derouane and the levitation effect summarized by Yashonath^23–26,43^ showed that confined diffusion is related to the ratio of molecule size and zeolite pore size. In their studies, several adjustable models were used to determine the relationship between molecular size (monoatomic or small molecules with less freedom) and diffusion behavior. It is noteworthy that due to the large freedom of long-chain alkanes (i.e., C12 in our work), molecular size is always varied in large ranges (i.e., larger than 6 Å, Supplementary Fig. 15) due to their great flexibility coupled with their rotational dynamics and unpredictable shape. In this work, the model of zeolite pore-confinement on the freedom and flexibility of long-chain alkane molecules has been proposed. In this case, the C12 dimension can be effectively manipulated to be strongly fixed in some special zeolites (see Supplementary Fig. 11). Consequently, the matching conditions of levitation diffusion have been achieved, and the goal of rapid diffusion is realized (Fig. 2). Our idea of improving diffusion by controlling molecular degrees of freedom is unique and has never been reported before but is of great significance to the zeolite diffusion mechanism. The effect of the concentration of the organic molecules and also the interaction with the acid sites of the zeolites will be the subject of future studies.

In summary, the superfast diffusion of molecules in nano-channels similar to a hyperloop was modeled and successfully presented in zeolites. A unique effect of molecular diffusion dependent on the zeolite pore size was observed, where a suitable, smaller pore size led to larger diffusion coefficients for long-chain molecules (the main reactants in the fluid catalytic cracking process). This counterintuitive behavior was closely related to the diffusion paths inside the zeolite and the shapes of the guest molecules. Similar to a hyperloop, superfast movement of the long-chain molecules was only achieved if the molecule maintained a linear structure and operated in the center of the channel. Under this condition, long-chain molecules had to remain linear and were distributed in the center due to the van der Waals forces of the channel. It is worth noting that diffusion slowed down considerably when the molecules deformed or deviated from the center orbits, causing frequent molecular collisions within the confined framework as the pore size increased. Moreover, uptake measurements with infrared microscopy experiments confirmed the hyperloop-like diffusion under this confinement. This work provided a unique perspective for the diffusion of molecules in zeolites, further revealed the microscopic mechanism of long-chain molecule diffusion in confined environments, and provided a reference for the selection of high-efficiency zeolites in the industrial field and in corresponding experimental conditions.

## Methods

### Molecular dynamics simulation

In our simulations, the initial structures of TON, MTW, AFI, and VFI were obtained from the International Zeolite Associations (IZA) database^44^ and optimized by the GULP^45^ with SLC^46,47^ core-shell force field. The 2 × 2 × 6, 2 × 10 × 4, 3 × 3 × 5, and 3 × 3 × 6 supercells with loadings of 8, 16, 9, and 9 alkane molecules (C4, C8, and C12) were used for TON, MTW, AFI, and VFI, respectively (one molecule per straight channel on average). For methane C1, four times the number of C12 molecules in each zeolite were used to avoid larger fluctuations in temperature. MD simulations were performed using the canonical ensemble (NVT), where the number of particles (*N*), volume (*V*), and temperature (*T*) were kept constant. The simulated temperatures were 298, 473, and 773 K, as controlled by the Nosé–Hoover thermostat with a coupling time constant of 0.1 ps. The leapfrog Verlet algorithm was used to integrate Newton’s equations of motion with a time step of 0.5 fs. The TraPPE-zeo^48^ and TraPPE-UA^49^ force fields (Supplementary Table 2) were used for zeolite and hydrocarbon, respectively. All Lennard–Jones cross-interaction parameters were determined by the Lorentz–Berthelot mixing rules, where the cutoff radius was 14 Å. Each MD simulation was equilibrated at 2 × 10^6^ steps, and then, 4 × 10^7^ production steps were recorded for research on the diffusion behaviors of adsorbate molecules. The trajectories were recorded every 1000 steps, and three independent MD simulations were conducted for better statistics. All MD simulations were performed using the DL_POLY 2.0 code^50^. Zeolite and molecule structure were visualized using the Materials Studio 7.0 software^51^.

### Experiments

The synthesis procedures of the TON, MTW, and AFI zeolites were obtained from existing methods in the literature^52,53^. The crystalline and phase purity of the samples were characterized by powder X-ray diffraction (pXRD) on a PANalytical X’Pert PRO X-ray diffractometer, where Cu Kα radiation (λ = 0.154059 nm) was used as the X-ray source, operated at 40 mA and 40 kV. The XRD patterns were recorded in the range of 2θ = 5–50°, and the morphologies and crystal sizes of the zeolites were observed by an FE-SEM Hitachi SU8020. The uptake rate of n-C_12_H_26_ was measured by an in situ infrared microscope (IRM). The zeolite samples were added to the chamber of the homemade in situ cell and dehydrated at 623 K for at least 2 h until no water was present in the infrared (IR) spectra. Then the steam of n-C_12_H_26_ vapor was introduced into the cell by nitrogen at 298 K. Meanwhile, the changes in the IR spectra of multiple crystal owning to the uptake of n-C_12_H_26_ at 298 K were recorded in real-time by a spectrometer (Bruker vertex 70 v). Integral IRM mode (1350–1620 cm^−1^) was used to obtain the uptake curves of n-C_12_H_26_ in the multiple-crystal forms of the TON, MTW, and AFI zeolites. The uptake rate measurements of n-C_4_H_10_ at 298 K (0 to 4 mbar) were performed using the Intelligent Gravimetric Analyzer (IGA-100, Hiden Analytical). Furthermore, the weight was achieved at a pressure of 10^−6^ mbar and a temperature of 623 K for at least 6 h. Then probe gas was introduced into the system with a carefully controlled quantity to ensure the isobaric and isothermal process. Meanwhile, the mass change, system pressure, and sample temperature were recorded in real-time. Nitrogen adsorption-desorption was conducted with a Micromeritics ASAP 2020 instrument at 77 K after the sample was degassed at 623 K under a vacuum for 6 h. The adsorption-desorption isotherms exhibited step uptakes near the ratio of P to P_0_ equal to 0 due to the microporous structure.

### Supplementary information

Supplementary information

Description of Additional Supplementary Files

Supplementary Movie 1

Supplementary Movie 2

Supplementary Movie 3

Supplementary Movie 4

### Source data

Source Data file

## Data Availability

The data of 3D drawing are provided in the supplementary data, other data presented in this manuscript are available from the corresponding authors upon request. Source data are provided with this paper.

## Code availability

The code used in this manuscript is available from the corresponding authors upon request.
