# Supplementary material for: Hyperloop-like diffusion of long-chain molecules under confinement
Source: Nat Commun. 2023 Mar 28;14:1735. doi: 10.1038/s41467-023-37455-3 (PMC10050162; doi:10.1038/s41467-023-37455-3)
Supplement: Supplementary file 1 — Supplementary information [file 41467_2023_37455_MOESM1_ESM.pdf]

Supplementary Information

Hyperloop-like diffusion of long-chain molecules under confinement

Yuan et al.

Table of Contents

Supplementary Note 1: Models and methods

Supplementary Note 2: Supplementary Figure 1 to 15

Supplementary Note 3: Supplementary Table 1 to 3

Supplementary Note 4: Supplementary References

Supplementary Note 1:

Models and analysis

Based on van der Waals (vdW) interaction potential (Supplementary Fig. 1) analysis [1 2], it shows the minimum value was 3.95 Å, as well as the potential function firstly decreases and then increases with the increase of the distance (r) between the oxygen atom of zeolite and the guest molecule. The interactions between subject and object is calculated by equation (1) [3]

$$u(r)=\sum_{i=1}^{N}4\varepsilon\left(\sigma^{12}/r_i^{12}-\sigma^{6}/r_i^{6}\right) \quad (1)$$

Corresponding areas of attraction (blue) and repulsion shown in Supplementary Fig. 1. In our simulations, all the nano-channel models were composed of oxygen atoms. Channel poresize were selected ranging from 7 to 12 Å, the lattice of each model were controlled a = b = 60 Å, c = 36.24 Å, α = β = γ= 90 °. Each model owns 16 channels with 1 molecule each channel.

Diffusion coefficient

In this work, the mean square displacement (MSD) of adsorbates is defined via the following equation (2)： [4]

$$MSD(\tau)=\frac{1}{N_m}\sum_{i}^{N_m}\frac{1}{N_\tau}\sum_{t_0}^{N_\tau}[r_i(t_0+\tau)-r_i(t_0)]^2 \qquad (2)$$

where N_m represents the number of gas molecules, N_τ is the number of time origins used in calculating the average, and r_i is the coordinate of the i-th molecule. In addition, the slope of the MSD as a function of time determines the self-diffusion coefficient (D_s) defined according to the Einstein relationship (equation (3))[1].

$$MSD(\tau) = 2nD_s\tau + b \qquad (3)$$

In which, n is the dimension of frameworks (n = 1 for 1-D diffusion). The diffusion coefficients were calculated by fitting the linear region of MSD using a least-square fit. The D_s values were obtained as the average of three dependent MD trajectories.

The influence of active site did not take into account in this work, studies have shown that the acidity of zeolite contributes the diffusion of alkenes in zeolites, but makes little influence on alkanes [5]. Furthermore, The flexibility of zeolite framework is also considered, and the conclusion is consistent with that of rigid one (Supplementary Table 3).

Non-bonded interactions [1]

In this paper, the guest−host interactions are described by pairwise-additive LJ (Lenard-Jones) 12−6 term, as following:

$$U(r) = 4\varepsilon[(\frac{\sigma}{r})^{12} - (\frac{\sigma}{r})^{6}] \quad (4)$$

Where r is the distance between two atoms, ε is LJ well depth, which reflects the strength of the interaction between two atoms, and σ is LJ diameter means the distance between atoms when the potential energy is equal to zero [6]. This is consistent with the parameter setting of force field above-mentioned (Fig. 3 a-h and Supplementary Fig. 1).

Density map

The density map (Supplementary Fig. 4, 9 and 10) is defined as

$$P = a \div b \times 100 \quad (5)$$

Where a is the number of C atoms in each gird (the radial plane of the zeolite channel is divided into grids) during the whole MD simulation time, and b is the number of total C atoms present over the simulation time.

Interaction energy [7]

The interaction energy (preferred adsorption sites and interaction energy barriers, Supplementary Fig. 12) is strongly correlated with the zeolite framework. Firstly, a C12 molecule was placed into the center of 1D channel in zeolite, then systematically moved from one end of the channel to the other following the diffusion path with 28 equi-spaced steps. The interaction energy between framework and molecule at each point was calculated, and the energy barrier for molecule diffusing in the center of 1D channel was determined by the difference between the lowest and highest energy along the diffusion pathway. All the interaction energy were calculated by the same force field and software of MD simulations.

Ab initio molecular dynamics (AIMD)

The periodic density functional theory (DFT) as well as the advanced AIMD simulation were performed using the CP2K [8-10] package. The TON, MTW and VFI frameworks were obtained from the IZA database [11]. The zeolite-dodecane (C12) complexes were cell optimized, and then, 30 ps AIMD simulations in NVT ensemble were carried out for zeolite-C12 complex at 298 K. A Nosé-Hoover [12] thermostat with a time constant of 100 fs and a time step of 0.5 fs was employed during the AIMD process. All the calculations were carried out using the Perdew-Burke-Ernzerhof (PBE) exchange-correlation functional [13] in conjunction with Grimme's D3 correction [14] with zero damping to account for dispersion interactions. The DZVP basis set and GTH pseudo potentials [15] were chosen for all elements. The plane wave cutoff energy and relative cutoff were 650 Ry and 60 Ry, respectively.

Uptake measurements implemented by infrared microscope (IRM)

Using Eq. (6) to decouple the surface barriers from overall mass transport and calculate surface permeability [16].

$$\frac{m_t}{m_\infty}|^{\sqrt{t}\to 0} \cong \frac{\alpha}{l}(\sqrt{t})^2 \tag{6}$$

where m_t/m_∞, t , l and α is the relative uptake loading of guest molecules, the uptake time, and the half thickness of the plane sheet (i.e., characteristic length of the intracrystalline diffusion) and the surface permeability, respectively.

Based on the obtained surface permeability, using dual resistance model (DRM) [17] to fit uptake curves can calculate the intracrystalline diffusivity D,

$$\frac{m_t}{m_\infty} = 1 - \sum_{n=1}^{\infty} \frac{2L^2 \exp(-\frac{\beta_n^2 Dt}{l^2})}{(\beta_n^2 + L^2 + L)\beta_n^2}; \ \beta_n \tan\beta_n = L \tag{7}$$

where D and $L = \alpha l/D$ is the intracrystalline (transport) diffusivity and the ratio of characteristic time of intracrystalline diffusion to that of surface barriers.

Reduced density gradient (RDG) analysis

Confinement effect in zeolite can be assessed by qualitative method, the scatter plot of the reduced density gradient (RDG) in real space is an effective and widely used tool to visualize non-covalent interaction between adsorbates and zeolite [18]. A random frame during the molecular dynamic simulation was selected to plot the RDG scatter as well as structures and dominant intermolecular interactions for the description of the host-guest interaction between the adsorbate and framework. The non-covalent interaction was performed in the region with low density and the RDG is defined as[18]

$$s = \frac{1}{2(3\pi^2)^{1/3}} \times \left(\frac{|\Delta\rho(r)|}{\rho(r)^{4/3}}\right) \quad (8)$$

together with the electron density ρ, which is used to distinguish the interaction (covalent and non-covalent). Hessian can be used to distinguish bonded ($\lambda_2 < 0$) from non-bonded ($\lambda_2 > 0$) interaction for the sign of the second largest eigen-value (λ_2) of the electron density. This helps to distinguish different types of interactions ((λ_2)$\rho < 0$, strong intramolecular interaction; ((λ_2)$\rho \approx 0$, weak van der Waals (vdW) interaction; (λ_2)$\rho > 0$, strong repulsive interaction). To reveal the confinement effect more preciously, the inter-molecular interaction was adapted for the RDG analysis. The RDG function was calculated by Multiwfn [19].

Distribution of deformation angle [20]

The deformation angle of the molecule is calculated from the deviation of the angle along the (i-2)th, (i)th and (i+2)th carbon atoms from equilibrium angle 180°. For example, there are eight (*i.e.,* n-4: n = 12 for dodecane) different angles (*i.e.,* 1-3-5, 2-4-6, 3-5-7, 4-6-8, 5-7-9, 6-8-10, 7-9-11, 8-10-12) for a dodecane molecule. As is depicted in Supplementary Fig. 3, the deviation of the angle ∠1 is following equation (9), where ∠2 represents the variable angle for the (i-2)th, (i)th and (i+2)th carbon atoms. Then, the number of occurrences for a maximal ∠1 of each molecule at various temperatures is counted, which indicates the degree of molecular bending and reflects the flexibility of the molecule in the confined channel at different temperatures.

$$\angle 1 = |180° - \angle 2| \quad (9)$$

Supplementary Note 2:

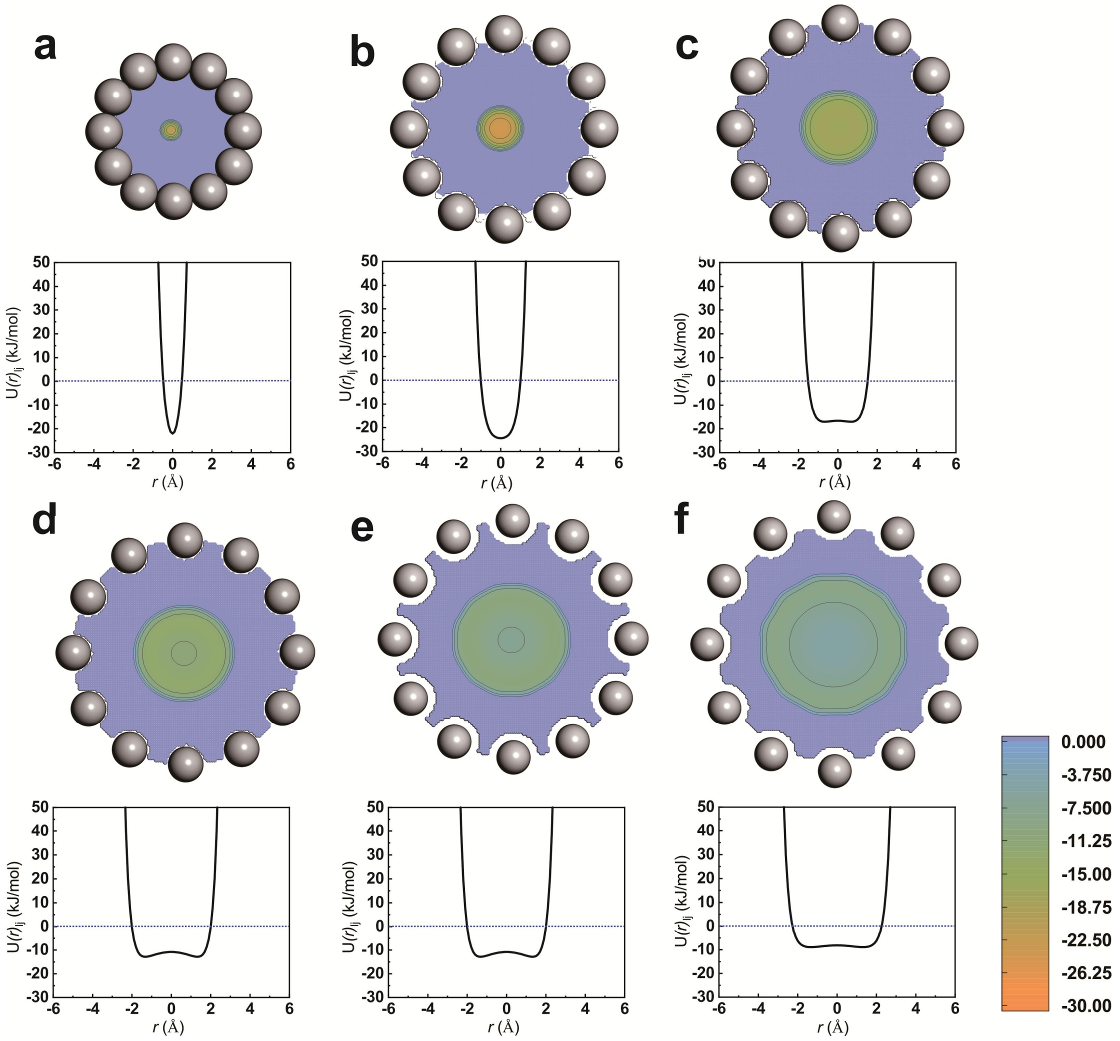

Supplementary Fig. 1| Van der Waals interactions. Areas of attraction (blue-green-orange) and repulsion (purple) in sub-nano models with different poresize (**a-f** represents 7-12 Å) calculated by the Lenard-Jones potential function. The channel with small poresize (7-8 Å) owns only one strongest adsorption site (minimal value in the center of the channel), while there are two strongest sites in the channel with larger poresize. Therefore, in analogy with the hyperloop, to keep the molecules in the center of the channel, 7-8 Å poresize seemed be the better. In addition, since molecules are not simple particles and have certain shapes (about 2 Å for linear alkane). If the poresize is too small, diffusion will be hindered by the confined space (molecules shown in purple areas, for the channel with a 7 Å poresize, only 1 Å for alkane to motion, while 2 Å for the channel with 8 Å). However, in the zeolites with large poresize, it will lead to rollover and diffusion slowed down as well. Therefore, the most suitable size should be the pore size where the attractive region and molecular size are matched (8 Å).

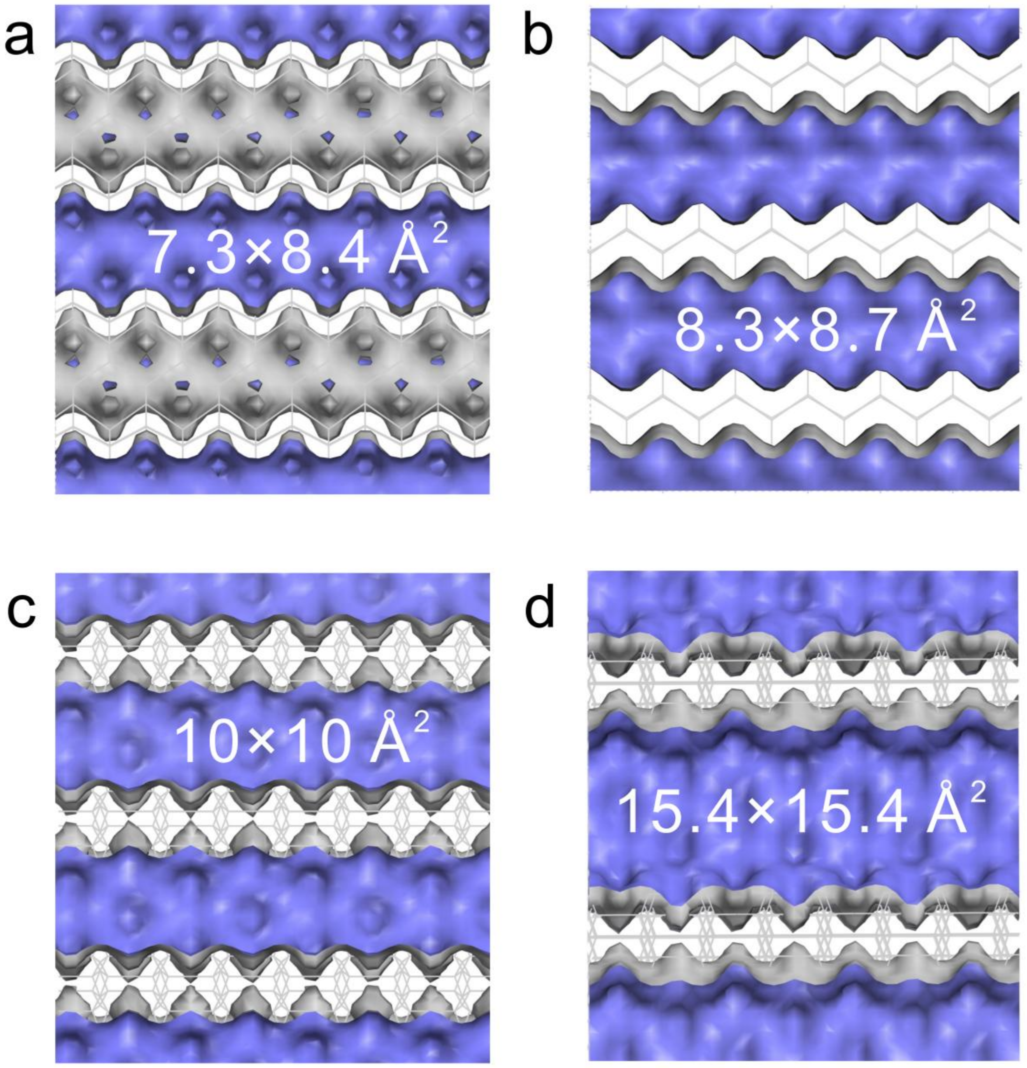

Supplementary Fig. 2| Channel structures of zeolites. The channel structure and poresize (The van der Waals radius of the O atom is not taken into account) of 1-D **a-d**, TON, MTW, AFI and VFI zeolite respectively.

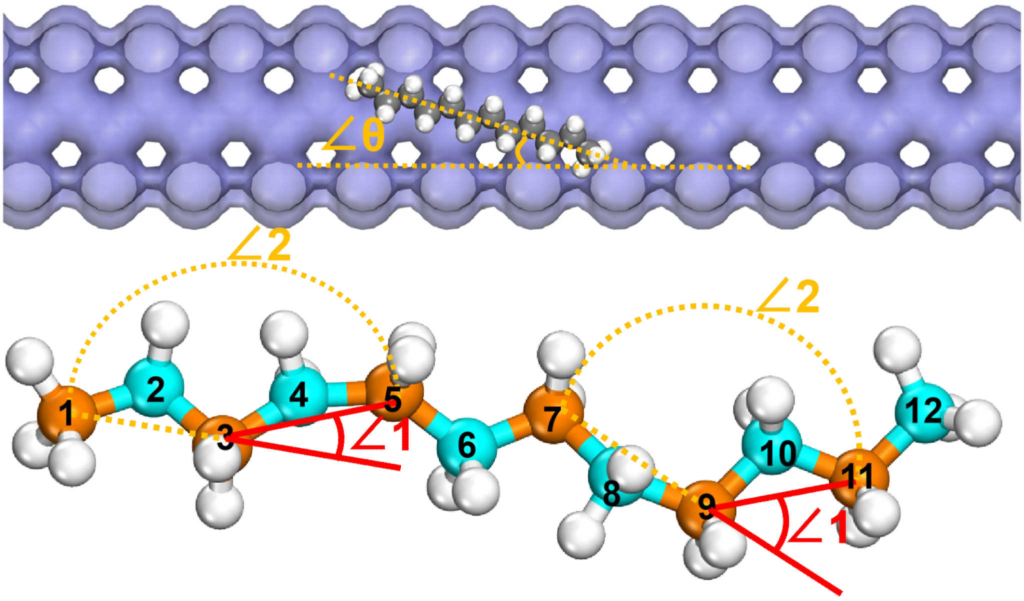

Supplementary Fig. 3| The schematic diagram of deviation angle (upper) and deformation angle (lower). The deviation angle (∠θ) is defined as the angle between the axial line of the zeolite channel and the end to end line of the molecule.

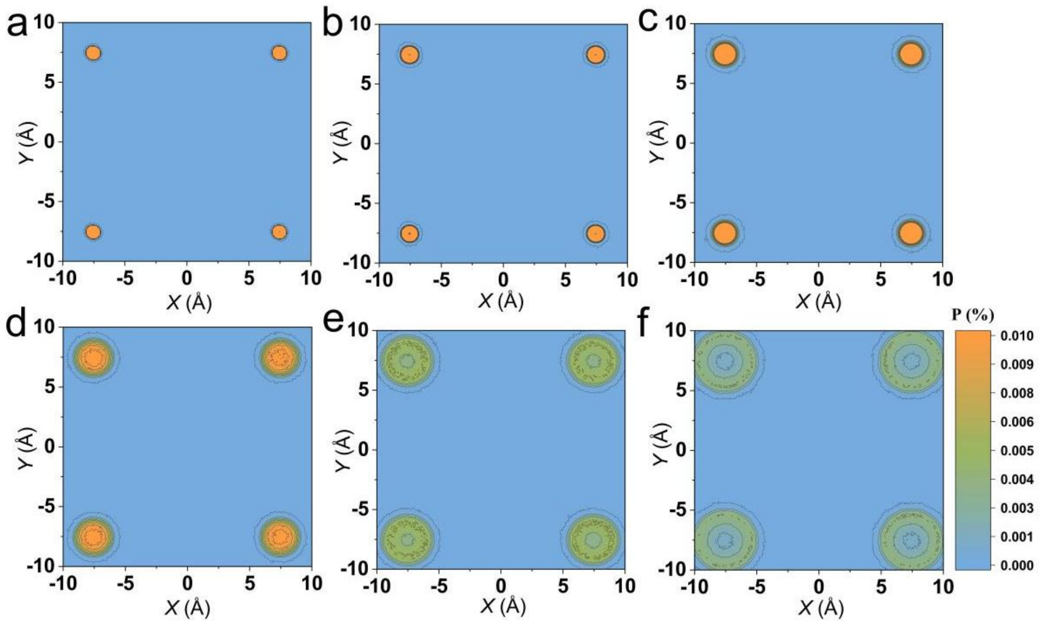

Supplementary Fig. 4| The density map of n-dodecane molecules at 298 K in the model of **a-f,** 7 to 12 Å channel model. It can be seen that with the increase of poresize, the probability of molecule distributed in the center of the channel decreases. As the poresize reaches 12 Å, it is found that molecules mainly located near the channel walls. It is completely consistent with the van der Waals analysis of Supplementary Fig. 1.

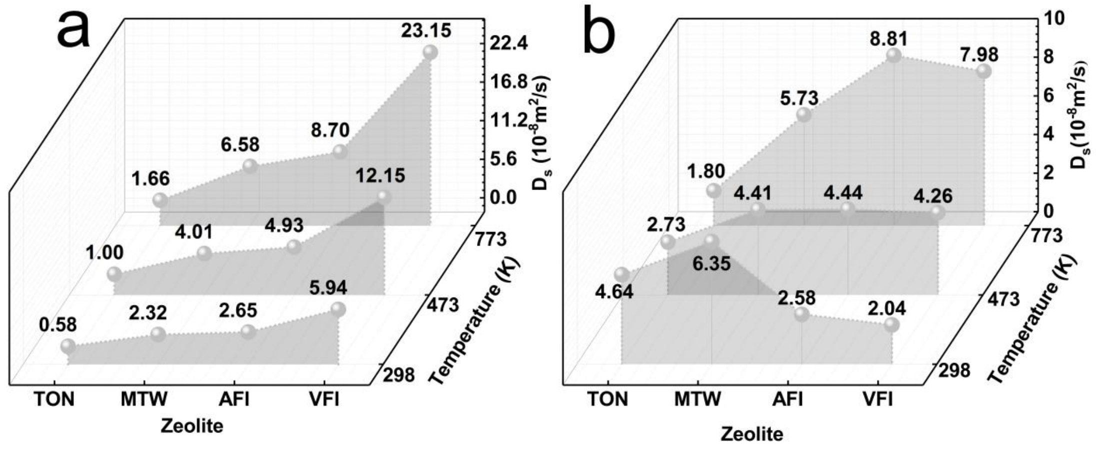

Supplementary Fig. 5| The diffusion coefficients of **a**, methane (C1) and **b,** n-octane (C8) at different temperatures in 1D channel zeolite. The diffusion coefficients (D_S) of C1 at 1-D channel zeolite possess the same trend of C4 (Fig. 2a), that the lager the poresize, the faster the diffusion, regardless of the temperature. While for C8, it is found that the D_s increases firstly and then decreases with the increase of zeolite poresize at 298 K. However, compared to AFI and VFI with larger poresize channel, the molecules in MTW and TON are difficult to flip over, and thus the diffusion coefficient first increases and then decreases.

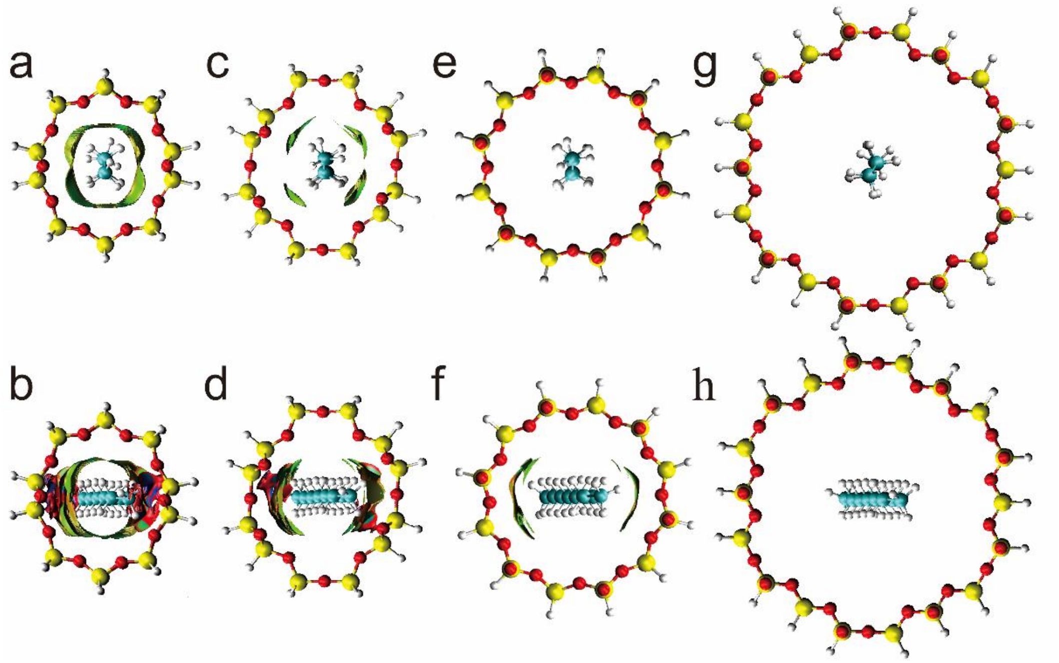

Supplementary Fig. 6| The reduced density gradient (RDG) of n-dodecane molecule with 0 (upper) and 10 (lower) degree deviation angle inside **a-b** TON, **c-d**, MTW, **e-f**, AFI and **g-h**, VFI zeolite. (The red, yellow, white and blue balls represent oxygen, silicon, hydrogen and carbon atoms respectively. The red part of the surface represents the strong repulsion, the green part is the vdw interaction, and the blue part is the strong adsorption).

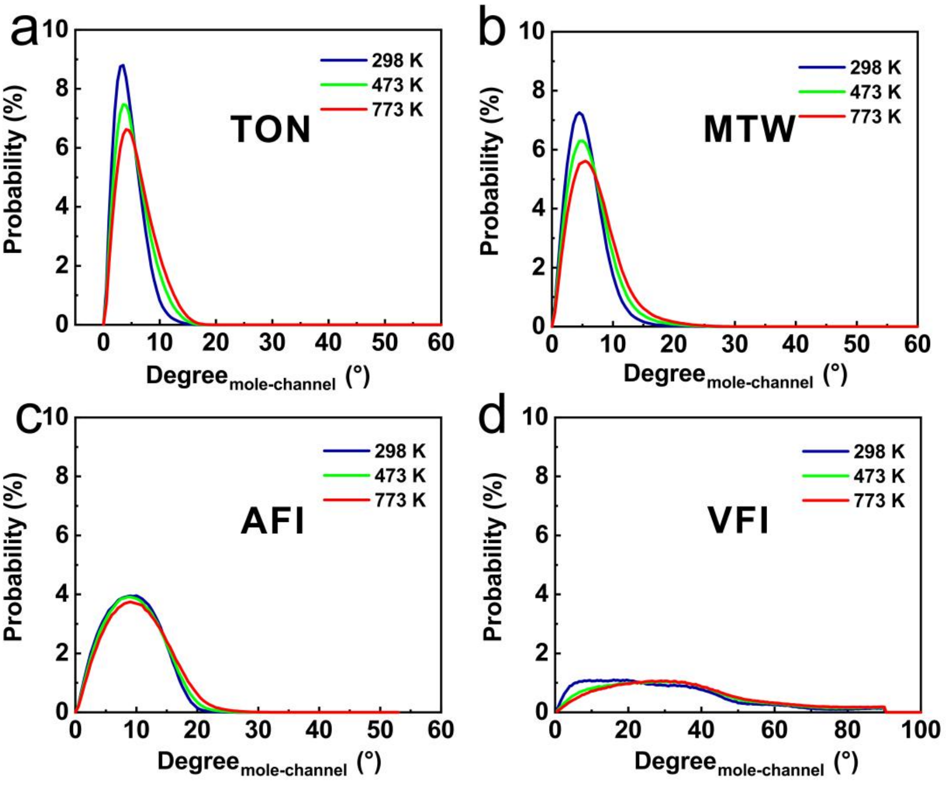

Supplementary Fig. 7| The deviation angle between the axial direction of zeolite channel and the end to end line of C12 molecule in 1D channel at different temperatures. It is found that a shift of deviation angle is shown as the temperature rising up regardless of poresize. This means that higher temperatures slight increase (less than 5 degrees) the deviation of the molecules from the axial direction of the channel, which indicates that there are other reasons for the presents of abnormal diffusion phenomena at high temperatures (the thermal resistance effect [20] mentioned in the text).

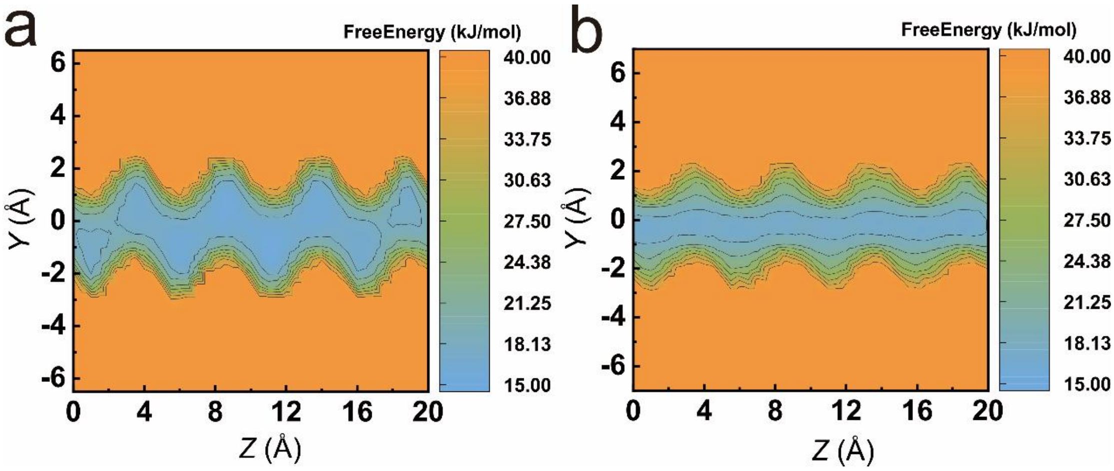

Supplementary Fig. 8| The free energy of **a**, C4 and **b**, C12 molecules in TON zeolite. The minimum of free energy of C4 and C12 respectively located in the curved position and in the center of the channel. Therefore, it is easier for C12 to diffuse in the center of the channel, while C4 prefers to transport near the pore wall.

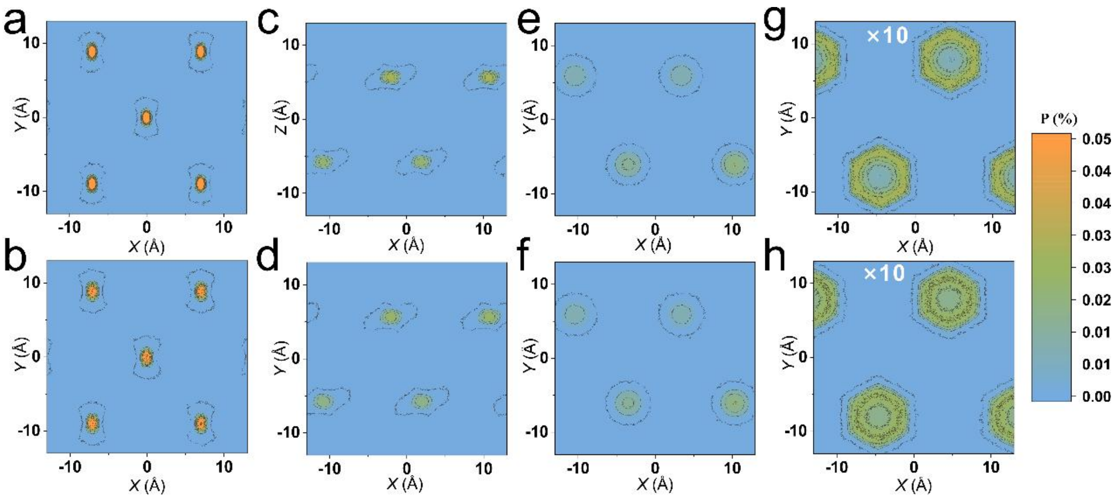

Supplementary Fig. 9| Density map. The probability that C atom distributed in the radial plane of channel of TON **(a-b)**, MTW (**c-d**), AFI (**e-f**), VFI (**g-h**) at 473 (upper) and 773 (lower) K. It can be seen that the distribution of C12 at high temperature is looser than that at low temperature (Fig. 3i-l), indicates molecules prefer to move deviating from the center of the channel at high temperature. Coupled with the thermal resistance effect (Fig. 4), therefore, this is no abnormality of the diffusion coefficient as pore size at high temperature.

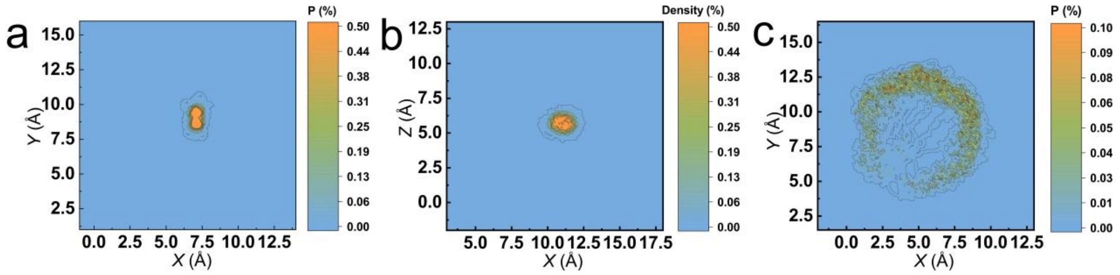

Supplementary Fig. 10| The density map of atom C of n-dodecane molecule in **a**, TON, **b**, MTW and **c**, VFI zeolite based on *ab initio* molecular dynamics simulation. It is obvious that the distributions of C atoms in TON and MTW is more concentrated than that in VFI with larger size, which is highly consistent with the results of classic MD calculations.

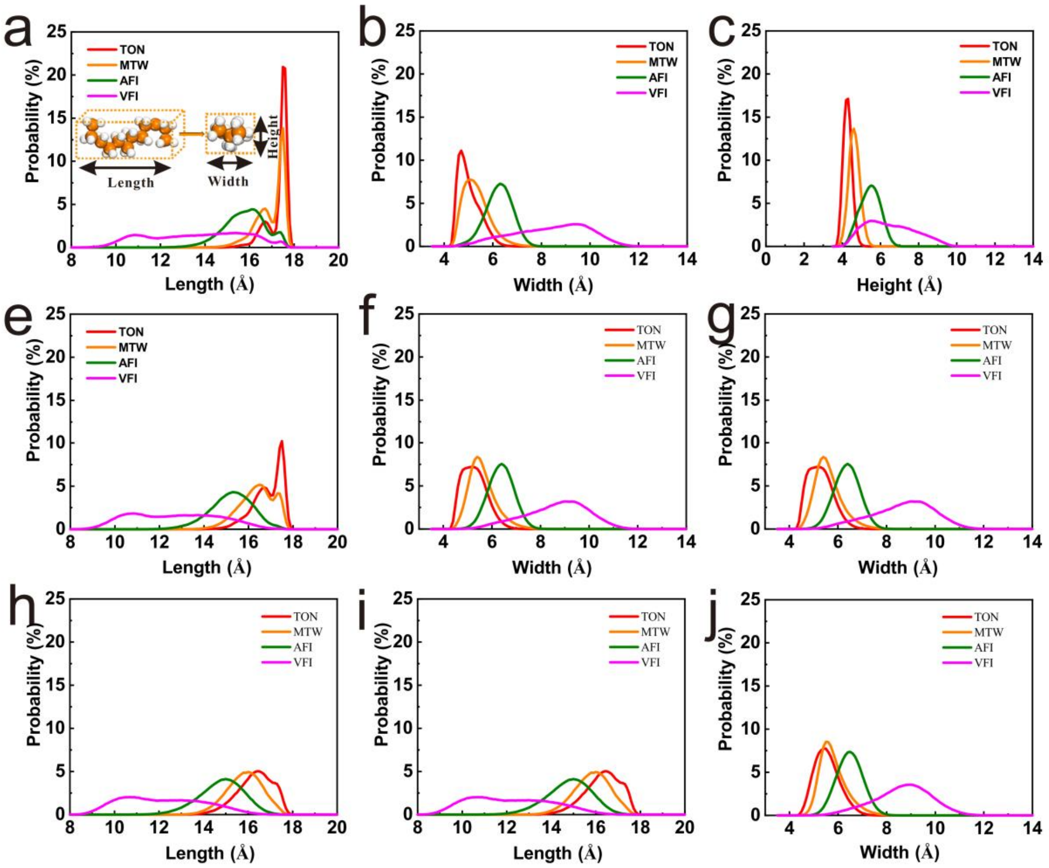

Supplementary Fig. 11| The shape of C12 molecules at various zeolites. The length (left), width (middle) and height (right) at 298 (a-c), 473 (e-g) and 773 K (h-j).

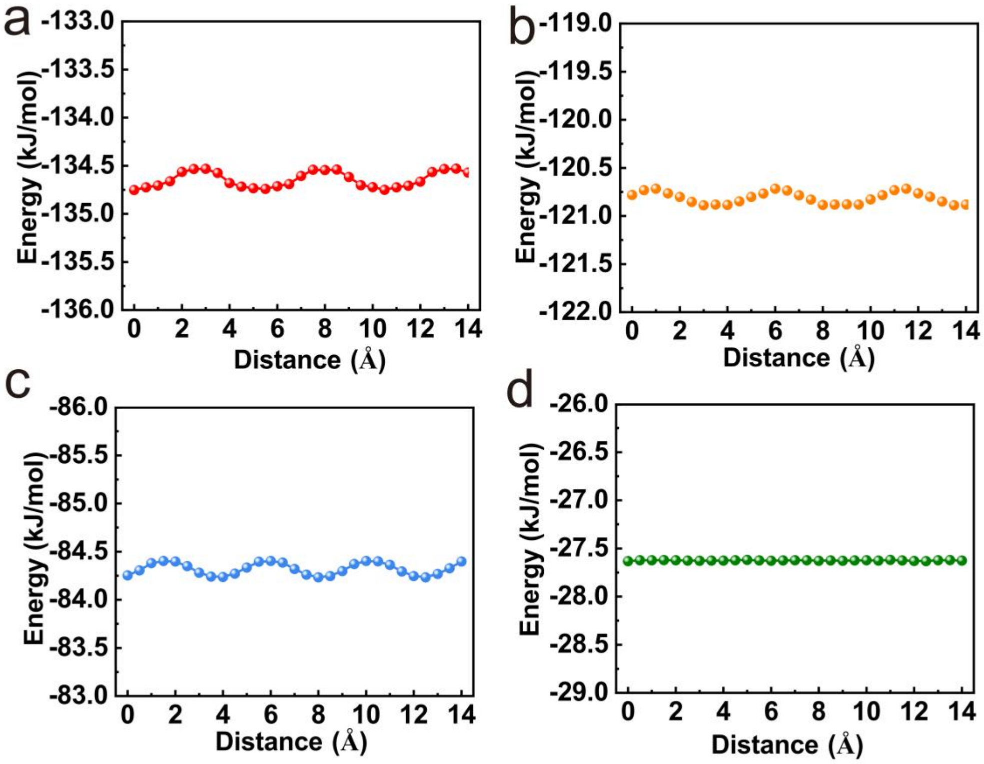

Supplementary Fig. 12| The interaction energy of dodecane molecule in the center of 1D channel TON, MTW, AFI and VFI (**a-d** respectively). When the molecules transport in the center of the zeolite channel and move along the axial direction, it can be seen that the energy barrier in all the 4 zeolites are very small.

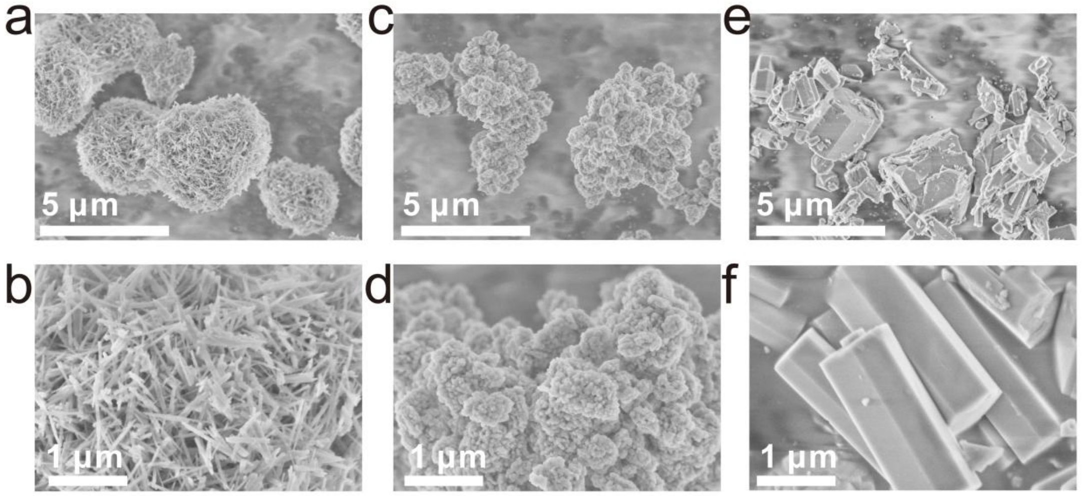

Supplementary Fig. 13| The scanning electron microscope (SEM) pictures of **a-b**, TON, **c-d**, MTW and **e-f**, AFI zeolite.

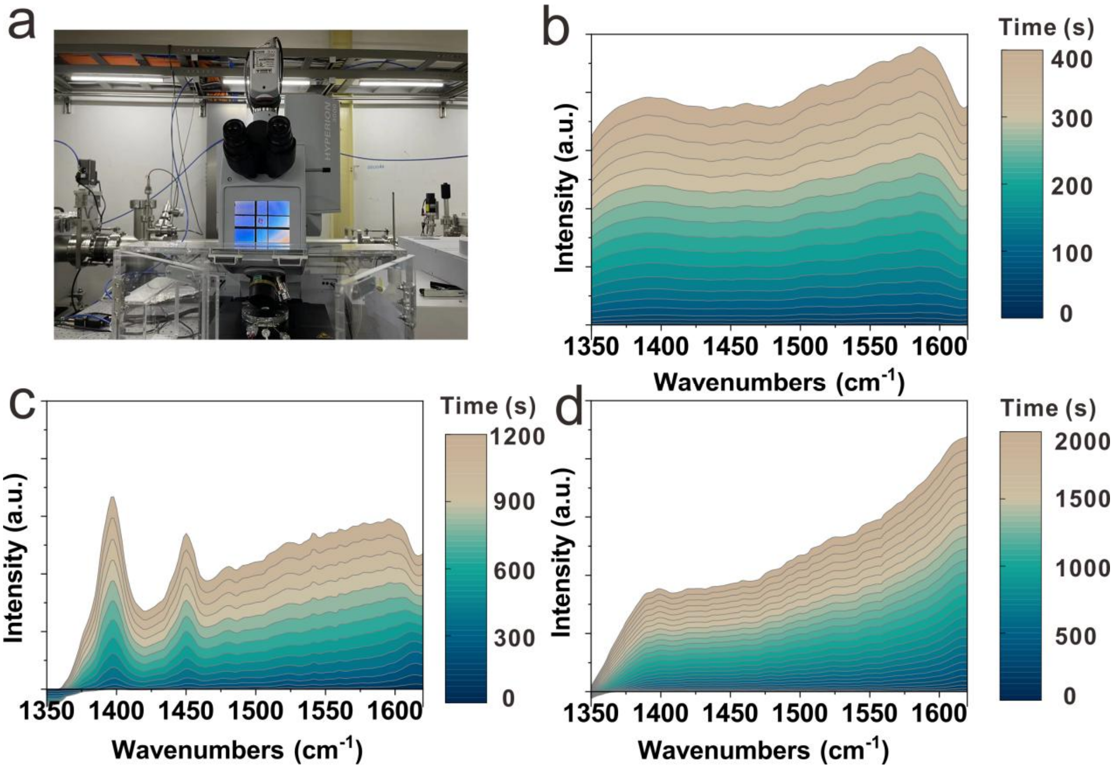

Supplementary Fig. 14| Infrared experiments. a, Implement of *in-situ* infrared microscope to the uptake rate measurements of n-$C_{12}H_{26}$ in zeolites; dynamic subtractive IR spectrum of n-$C_{12}H_{26}$ uptake within **b**, TON, **c**, MTW and **d**, AFI zeolites.

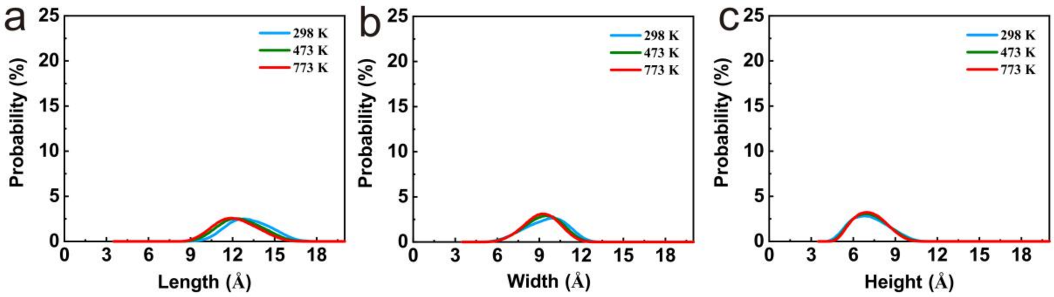

Supplementary Fig. 15| The shape of n-dodecane (a-c) in gas phase. The **a** length, **b** width and **c** height were calculated under various temperatures.

Supplementary Note 3:

Supplementary Table 1| Textural properties of AFI, MTW and TON zeolites with varying crystal sizes by N_2 adsorption and desorption isotherms at 77 K

Sample	Surface area (m^2/g)		Pore volume (cm^3/g)	
	S_{total}[a]	S_{micro}[b]	V_{total}[c]	V_{micro}[b]
AFI (H-SAPO-5)	320.86	294.95	0.17	0.15
MTW (H-ZSM-12)	205.14	187.51	0.18	0.14
TON (H-ZSM-22)	222.22	201.85	0.21	0.18

[a]BET surface area determined from multipoint method. [b]S_{micro} (micropore area) and V_{micro} (micropore volume) determined from the *t*-plot method. [c]V_{total} (total volume) is determined from adsorbed volume at p/p_0 = 0.98.

Supplementary Table 2| The force field used in the MD simulations, where the framework of zeolite were set as rigid.

van der Waals interactions (non-bonded interactions)			
Type	Potential	ε (kcal/mol)	σ (Å)
Si-Si	$U(r)=4\varepsilon\times[(\sigma/r)^{12}-(\sigma/r)^{6}]$	0.044	2.300
Si-O		0.068	2.800
Si-CH_3		0.092	3.025
Si-CH_2		0.063	3.125
O-O		0.105	3.300
O-CH_3		0.143	3.525
O-CH_2		0.098	3.625
CH_3-CH_3		0.195	3.750
CH_3-CH_2		0.133	3.850
CH_2-CH_2		0.091	3.950
CH_4-CH_4		0.294	3.730
Si-CH_4		0.113	3.015
O-CH_4		0.176	3.515

parameters for n-alkane (n>1)				
bonds	constraints	d = 1.54 (Å)		
angles	$U(\theta) = k\times(\theta-\theta_0)^2/2$	k = 124.197 (kcal/mol)	θ_0 = 114 (degree)	
dihedrals	$U(\varphi) =(A_1(1+\cos(\varphi)+A_2(1-\cos(2\varphi)+A_3(1+\cos(3\varphi))/2$	A_1 = 1.411 (kcal/mol)	A_2 = -0.271 (kcal/mol)	A_3 = 3.145 (kcal/mol)

Supplementary Table 3| The diffusion coefficients of n-dodecane molecules inside various zeolites with flexible framworks (the force field of flexible zeolite is the same as Demontis's work) [21].

D_s (10^{-10} m^2/s)			
TON	MTW	AFI	VFI
181.17	145.00	132.67	111.83

Supplementary Note 4:
Supplementary References:

1 Frenkel, D. & Smit, B. *Understanding molecular simulation: from algorithms to applications*. Vol. 1 (Elsevier, 2001).

2 Fasano, M. *et al.* Interplay between hydrophilicity and surface barriers on water transport in zeolite membranes. *Nat. Commun.* **7**, 8, doi:10.1038/ncomms12762 (2016).

3 Ghorai, P. K., Yashonath, S., Demontis, P. & Suffritti, G. B. Diffusion anomaly as a function of molecular length of linear molecules: levitation effect. *J. Am. Chem. Soc.* **125**, 7116-7123, doi:10.1021/ja028534i (2003).

4 Gao, S. S. *et al.* Cavity-controlled diffusion in 8-membered ring molecular sieve catalysts for shape selective strategy. *J. Catal.* **377**, 51-62, doi:10.1016/j.jcat.2019.07.010 (2019).

5 Cnudde, P. *et al.* Experimental and theoretical evidence for the promotional effect of acid sites on the diffusion of alkenes through small-pore zeolites. *Angew. Chem.-Int. Edit.* **60**, 10016-10022, doi:10.1002/anie.202017025 (2021).

6 Yashonath, S. & Santikary, P. Diffusion of sorbates in zeolites Y and A: Novel dependence on sorbate size and strength of sorbate-zeolite interaction. *J. Phys. Chem.* **98**, 6368-6376, doi:10.1021/j100076a022 (1994).

7 Liu, Z. Q. *et al.* Synergistically enhance confined diffusion by continuum intersecting channels in zeolites. *Sci. Adv.* **7**, 9, doi:10.1126/sciadv.abf0775 (2021).

8 VandeVondele, J. *et al.* Quickstep: Fast and accurate density functional calculations using a mixed gaussian and plane waves approach. *Comput. Phys. Commun.* **167**, 103-128, doi:10.1016/j.cpc.2004.12.014 (2005).

9 VandeVondele, J. & Hutter, J. Gaussian basis sets for accurate calculations on molecular systems in gas and condensed phases. *J. Chem. Phys.* **127**, 9, doi:10.1063/1.2770708 (2007).

10 Hutter, J., Iannuzzi, M., Schiffmann, F. & VandeVondele, J. CP2K: atomistic simulations of condensed matter systems. *Wiley Interdiscip. Rev.-Comput. Mol. Sci.* **4**, 15-25, doi:10.1002/wcms.1159 (2014).

11 Baerlocher, C. J. h. w. i.-s. o. d. Database of zeolite structures. (2008).

12 Martyna, G. J., Klein, M. L. & Tuckerman, M. Nose-hoover chains-the canonical ensemble via continuous dynamics. *J. Chem. Phys.* **97**, 2635-2643, doi:10.1063/1.463940 (1992).

13 Perdew, J. P., Burke, K. & Ernzerhof, M. Generalized gradient approximation made simple. *Phys. Rev. Lett.* **77**, 3865-3868, doi:10.1103/PhysRevLett.77.3865 (1996).

14 Grimme, S., Antony, J., Ehrlich, S. & Krieg, H. A consistent and accurate ab initio parametrization of density functional dispersion correction (DFT-D) for the 94 elements H-Pu. *J. Chem. Phys.* **132**, 19, doi:10.1063/1.3382344 (2010).

15 Goedecker, S., Teter, M. & Hutter, J. Separable dual-space gaussian pseudopotentials. *Phys. Rev. B* **54**, 1703-1710, doi:10.1103/PhysRevB.54.1703 (1996).

16 Gao, M. B. *et al.* Direct quantification of surface barriers for mass transfer in nanoporous crystalline materials. *Comm. Chem.* **2**, 10, doi:10.1038/s42004-019-0144-1 (2019).

17 Crank, J. *The mathematics of diffusion*. (Oxford university press, 1979).

18 del Campo, J. M., Gazquez, J. L., Alvarez-Mendez, R. J. & Vela, A. The reduced density gradient in atoms. *Int. J. Quantum Chem.* **112**, 3594-3598, doi:10.1002/qua.24241 (2012).

19 Lu, T. & Chen, F. W. Multiwfn: A multifunctional wavefunction analyzer. *J. Comput. Chem.*

33, 580-592, doi:10.1002/jcc.22885 (2012).

20 Yuan, J. M. *et al.* Thermal resistance effect on anomalous diffusion of molecules under confinement. *Proc. Natl. Acad. Sci. U. S. A.* **118**, 6, doi:10.1073/pnas.2102097118 (2021).

21 Demontis, P., Suffritti, G. B., Quartieri, S., Fois, E. S. & Gamba, A. Molecular dynamics studies on zeolites. 3. dehydrated zeolite A. *J. Phys. Chem.* **92**, 867-871, doi:10.1021/j100315a003 (1988).
