# Supplementary material for: Hyperloop-like diffusion of long-chain molecules under confinement
Source: Nat Commun. 2023 Mar 28;14:1735. doi: 10.1038/s41467-023-37455-3 (PMC10050162; doi:10.1038/s41467-023-37455-3)
Supplement: Supplementary file 2 — Description of Additional Supplementary Files [file 41467_2023_37455_MOESM2_ESM.docx]

**Description of Additional Supplementary Files**

Supplementary Movie 1

Description: Dynamic process of C12 molecules in TON zeolite at 298 K by molecular dynamics simulations.

Supplementary Movie 2

Description: Dynamic process of C12 molecules in VFI zeolite at 298 K by molecular dynamics simulations.

Supplementary Movie 3

Description: Dynamic process of C4 molecules in TON zeolite at 298 K by molecular dynamics simulations.

Supplementary Movie 4

Description: Dynamic process of C4 molecules in VFI zeolite at 298 K by molecular dynamics simulations.
